# Courbes de croissance fœtale non ajustées et personnalisées: une étude comparative dans une population du Sud du Sahara

**DOI:** 10.11604/pamj.2021.39.51.27307

**Published:** 2021-05-19

**Authors:** Abdoulaye Diakhaté, Mame Diarra Ndiaye, Mamour Guèye, Magatte Mbaye, Pape Moctar Faye, Moussa Diallo, Simon Birame Ndour, Mouhamadou Wade, Aliou Diouf, Jean Charles Moreau

**Affiliations:** 1 Clinique Gynécologique et Obstétricale de l´Hôpital Aristide Le Dantec, Université Cheikh Anta Diop de Dakar, Dakar, Sénégal,; 2 Centre de Santé Philippe Maguilèn Senghor, Université Gaston Berger de Saint-Louis, Saint-Louis, Sénégal,; 3 Hôpital d´Enfants Albert Royer, Université Cheikh Anta Diop de Dakar, Dakar, Sénégal

**Keywords:** Age gestationnel, Gardosi, EPOPé M0, courbes de croissance, Gestational age, Gardosi, EPOPé M0, fetal growth curves

## Abstract

Le but de notre étude était de comparer la courbe non ajustée EPOPé M0 à la courbe personnalisée de Gardosi, dans le diagnostic des fœtus petits pour l´âge gestationnel (PAG) au sein d´une population du Sud du Sahara. Les classements des courbes de Gardosi et al. et EPOPé M0 étaient comparée. Les différences de classement ont été analysées selon les caractéristiques des patientes et des pathologies obstétricales. Les données recueillies à partir d´un logiciel Filemaker étaient analysées à l´aide des logiciels SPSS 20.0 et R Studio. Les tests statistiques étaient réalisés selon les conditions d´applicabilité. Le risque d´erreur alpha était fixé à 0,05. Avec la courbe de Gardosi, le taux de nouveau-nés PAG était plus élevé (31,4% contre 28,9%) et le taux de PAG ne différait pas entre les femmes en surpoids et celles de poids normal. Le taux de PAG sévères chez les prématurés était aussi plus élevé (23,6 versus 19,7%). Les pathologies étaient plus fréquentes chez les nouveau-nés classés PAG sévères par la courbe personnalisée. La courbe personnalisée est plus adaptée dans la population d´Afrique Sub-saharienne.

## Abstract (en)

The purpose of this study was to compare the unadjusted EPOPé M0 curve with the customized Gardosi curve in the diagnosis of small-for-gestational-age (SGA) fetuses in a sub-Saharan population. We compared the Gardosi et al. and EPOPé M0 classifications. Classification differences were analyzed according to patient characteristics and obstetric conditions. Data collected from FileMaker software were analyzed using SPSS 20.0 and R Studio software. The statistical tests were carried out according to applicability conditions. Alpha risk was set at 0.05. The Gardosi curve showed that the rate of SGA newborns was higher (31.4% versus 28.9%) and did not differ between overweight and normal-weight women. The rate of severe SGA in preterm infants was also higher (23.6 versus 19.7%). Diseases were more frequent in newborns classified as severe SGA by the customized growth curve. The customized curve is recommended for the sub-Saharan Africa population.

## Introduction

Le fœtus ou nouveau-né petit pour l´âge gestationnel (PAG) est une situation fréquente avec une prévalence de 8% dans la population générale [1]. Il s´agit de fœtus ou nouveau-nés dont le poids ou les mensurations se situent en dessous du 10^e^ percentile pour l´âge [2]. Outre la mortalité et la morbidité périnatale inhérente à cet état, de récentes études l´ont identifié comme facteur de risque de pathologies métaboliques dans l´enfance et l´adolescence [3, 4]. Dans l´optique d´affiner leur dépistage et tenant compte de la notion de potentiel de croissance fœtale génétiquement programmée, certains auteurs ont proposé des courbes de croissance personnalisées en complément ou en alternative aux courbes de croissance dites standardisées [5-8]. Cette étude compare les classements diagnostiques de la courbe customisée de Gardosi *et al*. [6] à la courbe non ajustée EPOPé M0 [9] chez les PAG dans une maternité de niveau II du Sénégal.

## Méthodes

Il s´agissait d´une étude analytique, observationnelle, réalisée sur une cohorte rétrospective d´une durée de 2 ans: de janvier 2017 à décembre 2018. Tous les couples mères-nouveau-nés, enregistrés après accouchement dans le service durant la période d´étude, étaient inclus. La condition nécessaire à l´inclusion de chacun des couples était un enregistrement complet et précis des variables que sont: le terme de la grossesse calculé sur la base de la date des dernières règles ou d´une échographie réalisée entre 11 semaines d´aménorrhée (SA) et 13 SA 6 jours; le poids de la mère au début de la grossesse; la taille de la mère; la parité; le poids et le sexe du nouveau-né.

Les situations cliniques que sont les grossesses multiples, les malformations fœtales, les anomalies de la différenciation sexuelle et les morts fœtales *in utero* n´étaient pas incluses dans l´étude. Les données étaient recueillies à partir d´un logiciel *Filemaker Pro Advanced 16* [10]. Les équipes procédaient à un enregistrement quotidien des dossiers cliniques. Nous avions étudié le profil démographique et biométrique de la mère: parité, âge, poids, taille et indice de masse corporelle (IMC). Les données relatives au nouveau-né étaient également étudiées: poids, score d´Apgar, terme de gestation et mortalité *per partum*. Enfin, nous avions étudié l´incidence de certaines pathologies de la grossesse telles que: l´hypertension artérielle, l´hématome rétroplacentaire et le diabète. Ces paramètres avaient permis de classer chaque nouveau-né selon la courbe de Gardosi et EPOPé M0 puis d´analyser les différences. Gardosi *et al*. ont initié en 1992, au Royaume-Uni, un concept de poids de naissance attendu basé sur: le modèle de croissance in utero proposé initialement par Hadlock *et al*. et ajusté sur des paramètres constitutionnels (taille et poids de la mère en début de grossesse, parité, ethnie, ainsi que sexe du fœtus).

Chacun de ces paramètres sus-mentionnés ont un effet significatif et indépendant sur le poids de naissance [11]. Le modèle EPOPé M0 non ajusté, prédit au 50^e^ percentile à 40 + 0 SA un poids de 3387,6 g. Les équations de calcul du poids aux 3^e^, 10^e^, 503^e^, 903^e^ et 973^e^ percentiles sont décrites dans l´^9^]. Les données recueillies étaient analysées à l´aide des logiciels SPSS 20.0 et R Studio version 1.1.383.51. Nous avions étudié les distributions des variables quantitatives et calculé les fréquences des données qualitatives. Les tests statistiques étaient réalisés selon les types de variables, la taille de l´échantillon et des sous-échantillons. Afin de comparer les différences de classement de la population dans les différents modèles, nous avions utilisé les tests non paramétriques pour échantillons appariés, notamment ceux de McNemar et de Q Cochran. Dans tous les cas le risque d´erreur de première espèce alpha était fixé à 0,05. Enfin, sur la base de la morbidité et de la mortalité néonatales précoces, nous avions calculé la sensibilité et la spécificité des deux modèles. Les nouveau-nés dont le poids de naissance était inférieur au 10^e^ percentile définissaient les PAG, il s´agissait de PAG sévère lorsque le poids était inférieur au 3^e^ percentile. Les cas discordants correspondaient aux nouveau-nés classés PAG par une courbe et eutrophes par la courbe de comparaison.

## Résultats

Durant la période d´étude, étaient inclus 2419 couples mère/nouveau-né. Les caractéristiques maternelles et néonatales décrivant la population d´étude sont résumées dans le Tableau 1. Près de la moitié de la population avait un IMC supérieur ou égale à 25 Kg/m^2^. Les proportions de nullipares et multipares étaient sensiblement les mêmes. Avec la courbe de Gardosi *et al*. Un peu plus de trente-et-un (31,4)% des nouveau-nés étaient PAG. En utilisant le modèle EPOPé M0, ce taux était de 28,9%. Une différence significative de 2,5% était mise en évidence (Tableau 2). D´après le test de Q Cochran, une différence significative étaient notée également dans la répartition des PAG sévères et modérés (Tableau 3). Selon le modèle de Gardosi *et al*. le taux de PAG était similaire chez les patientes en surpoids et chez celles avec un IMC inférieur à 25 Kg/m^2^. Par contre, selon EPOPé M0, on retrouvait moins de nouveau-nés PAG chez les patientes en surpoids. Une différence statistiquement significative de 12,7% était notée (Tableau 4).

**Tableau 1:** distributions des différentes caractéristiques des mères et nouveau-nés dans la population d´étude

Variables Distributions	Moyenne (écart type)	Médiane	Pourcentage (%)
**Age (ans)**	27,3 (6,2)	27,0	--
**Poids (aKg)**	69,3 (14,8)	69,0	--
**Taille (bcm)**	165,0 (7,0)	162,0	--
**cIMC (dKg/m2)**	25,4 (5,1)	24,6	
**<25**			54,0
**≥ 25**			46,0
**Parité**	1,1 (1,4)	1,0	--
**Nullipares**			47,1
**Multipares**			52,9
**Terme (eSA)**	39,3 (1,9)	39,0	
**Prématurité**			5,8
**Terme**			94,2
**Poids de naissance**	3142 (525,1)	3100	--
**Score d´Apgar fM5**			
**≥ 7**	--	--	87,5
**<7**	--	--	12,5
**Sexe**			
**Masculin**	--	--	47,7
**Féminin**	--	--	52,3

aKg= Kilogramme, bcm= centimètres cIMC= Indice de Masse Corporelle, dKg/m2= Kilogramme par mètre carré, eSA= semaines d´aménorrhée, fM5= 5^e^ minute

**Tableau 2:** répartition des nouveau-nés au 10^e^ percentile selon les courbes de Gardosi *et al*. et EPOPé M0

Courbes	<10^e^ percentile	≥ 10^e^ percentile	Total	p
	n (%)	n (%)	n (%)	
Gardosi *et al*	759 (31,4)	1660 (68,6)	2419 (100)	0,000
EPOPé M0	698 (28,9)	1721 (71,1)	2429 (100)	

**Tableau 3:** répartition des nouveau-nés aux 3^e^ et 10^e^ percentiles selon les courbes de Gardosi *et al*. et EPOPé M0

Courbes	< 3^e^ percentile n (%)	3-10^e^ percentiles n (%)	≥ 10^e^ percentile n (%)	p
Gardosi *et al*.	383 (15,8)	376 (15,5)	1660 (68,7)	0,000
EPOPé M0	342 (14,1)	344 (14,2)	1733 (71,6)	

**Tableau 4:** comparaison de la classification des nouveau-nés par les courbes de Gardosi *et al*. et EPOPé M0 en fonction de l´indice de masse corporelle

aIMC (bKg/m)	Effectifs (n)	Pourcentages (%)	p-value
		**EPOPé M0**	
≥ 25	245	22,0%	0,000
< 25	453	34,7%	
Gardosi *et al*			
≥ 25	345	31,0%	0,382
< 25	414	31,7%	

aIMC= Indice de Masse Corporelle, bKg/m= Kilogramme par mètre carré

Le modèle Gardosi *et al*. classaient moins d´enfants prématurés PAG (30% contre 32,2%). La différence était statistiquement significative. Cependant le taux de PAG sévères était plus élevé avec le modèle de Gardosi *et al*. (23,6 versus 19,7%) (Tableau 5). Chez les mères diabétiques, l´incidence des PAG sévères était plus élevée avec la courbe de Gardosi *et al*. (14,6 versus 8,7%). Le constat était le même pour les parturientes dont la grossesse était compliquée par une hypertension artérielle ou un hématome rétroplacentaire (28,4 contre 26,6% (Tableau 6). En comparant les modèles Gardosi *et al*. et EPOPé M0, les nouveau-nés discordants étaient au nombre de 255. Les pathologies étaient plus fréquentes chez les nouveau-nés classés PAG uniquement dans le modèle de Gardosi *et al*. (Tableau 6). Cependant, du fait de la taille de certains sous-échantillons, la différence n´était pas toujours significative. En tenant compte de la morbidité et de la mortalité néonatale précoces, le modèle de Gardosi *et al*. avaient une sensibilité de 37% et une spécificité de 70% pour classer des nouveau-nés PAG. Concernant le modèle d’EPOPé M0, ces paramètres étaient respectivement de 35 et 72%.

**Tableau 5:** répartition des nouveau-nés en fonction des courbes et des pathologies maternelles et obstétricales

Courbes	<aP3 n (%)	P3-P10 n (%)	≥bP10 n (%)	p
		**Diabète**		
**Gardosi *et al*.**	15 (14,6)	15 (14,6)	73 (70,8)	0,510
**EPOPé M0**	9 (8,7)	14 (13,6)	80 (77,7)	0,036
**Hypertension artérielle et hématome rétroplacentaire**				
**Gardosi *et al*.**	63 (28,4)	33 (14,9)	126 (56,7)	0,000
**EPOPé M0**	59 (26,6)	27(12,2)	136 (61,2)	0,000
		**Prématurité**		
**Gardosi *et al*.**	33 (23,6)	9 (6,4)	98 (70)	0,000
**EPOPé M0**	25 (17,9)	20 (14,3)	95 (67,8)	0,008

aP3= 3^e^ percentile, bP10= 10^e^ percentile

**Tableau 6:** comparaison de différents paramètres au sein des nouveau-nés discordants

Paramètres	Gardosi 10^e^ p	EPOPé M0 <10^e^ p	p-value
Répartition	158 (62%)	97 (38%)	0,000
aHTA et bHRP	18 (64,3%)	10 (35,7%)	0,480
Diabète	7 (77,8%)	2 (22,2%)	0,266
Score d´Apgar<7	13(46,4%)	15(53,6%)	0,058
Mortalité per partum	3 (75%)	1 (25%)	0,509
IMC>25	112 (90,7%)	12 (9,2%)	0,000

aHTA: Hypertension artérielle, bHématome rétro-placentaire.

## Discussion

### Age et PAG

Des études récentes se sont intéressées à l´association entre âge maternel et faible poids de naissance [2, 11-13]. Kangulu *et al*. concluent à un risque de FPN plus élevé chez les femmes de moins de 18 ans (OR =7,62) et de plus de 35 ans (OR = 2,04) [13]. Sauer *et al*. à New York, calculent un *OR* significatif à 1,53 chez les femmes de plus de 44 ans [2]. Ce risque identifié chez les gestantes d´âge élevé est lié aux multiples complications de la grossesse que sont, entre autres, la prééclampsie, le diabète gestationnel et les aneuploïdies [2]. L´intérêt que suscite ce sujet est lié à la fois au recul observé de l´âge moyen à la maternité mais aussi aux disparités observées entre les régions [14].

### Comparaison des courbes EPOPé M0 et Gardosi et al

A travers des tests non paramétriques, une différence statistiquement significative était mise en évidence dans le classement des modèles de Gardosi et d´EPOPé M0. La prise en compte des facteurs maternels et du sexe conduit à classer 2,5% d´enfant PAG en plus. Selon Ego *et al*. la prise en compte des paramètres maternels conduit à reclasser 4,0% des naissances par les différents modèles EPOPé [9]. Une étude multinationale de Francis *et al*. [15] a comparé, en 2018 le modèle *INTERGROWTH-21^st^* (IG21) à une courbe nommée *Gestation-related optimal weight (GROW)* ajustée au poids, à la taille, à la parité et à l´origine ethnique de la mère. Cette étude démontre d´abord qu´en utilisant IG21, il existe de grandes différences des taux de PAG au sein des 10 cohortes étudiées, allant de 3,1 à 16,8%. Le poids optimal calculé avec le logiciel *GROW* suggère que les taux de SGA varient principalement en raison de différences physiologiques entre les différentes populations. En population française, Ego *et al*. ont mis en évidence un taux de PAG chez les prématurés variant de 21,9 à 22,3% entre les modèles EPOPé M0 et M1 [9].

Dans notre échantillon, le modèle de Gardosi *et al*. classe moins d´enfants prématurés PAG que la courbes EPOPé M0. Cependant, on compte selon ce modèle plus de PAG sévères (inférieur au 3^e^ percentile). Il est reconnu que la prématurité est associée à la naissance d´un enfant PAG du fait de la pathologie l´ayant induite. Le surpoids correspond à un IMC compris entre 25,1 et 29,9 Kg/m^2^ et l´obésité à un IMC supérieur ou égal à 30 Kg/m^2^ [16]. La prévalence de l´obésité, en constante augmentation dans le monde, en fait une pandémie. La surcharge pondérale est donc une situation fréquente au cours de la grossesse qui expose à des complications maternelles (diabète gestationnel, hypertension artérielle) et fœtales (macrosomie). L´hypertension artérielle est associée à un risque accru de retard de croissance intra-utérin (RCIU) [17].

Dans l´échantillon d´étude, les courbes EPOPé M0 classent moins de nouveau-nés PAG chez les femmes obèses et en surpoids que chez les mères dont l´IMC est inférieur à 25 Kg/m^2^ (22% contre 34,7%) avec une différence statistiquement significative. En utilisant le modèle de Gardosi *et al*. les proportions de PAG ne diffèrent pas dans les 2 groupes. Selon Maisonneuve *et al*. l´incidence de la restriction de croissance intra-utérine est généralement moindre chez les femmes obèses si l´on exclut les femmes présentant des pathologies hypertensives [16]. Ces différences, notées dans notre échantillon, suggèrent une probable sous-estimation des PAG dans la population en surpoids par les courbes standardisées. Galtier-Dereure *et al*. retrouvent une fréquence d´hypertension artérielle (HTA) multipliée par 3,6 à 3,7 chez les patientes en surpoids et par 2,2 à 21,4 chez les femmes en obésité. Toutefois, ils précisent que ces états hypertensifs ont un faible retentissement sur la croissance fœtale, puisque l´incidence des RCIU chez les patientes souffrant d´obésité est inférieure de moitié à celle retrouvée chez les groupes témoins [17]. Si les modèles standards sont utilisés, la prévalence des PAG pourrait alors bien être sous-estimée. L´indice de masse corporelle était supérieur ou égal à 25 Kg/m^2^ pour près de la moitié de la population d´étude (46%). Il est important d´utiliser en plus des courbes standardisées, celles personnalisées afin de ne pas méconnaître dans cette importante partie de la population, des fœtus PAG, ce qui est un facteur de morbidité supplémentaire. Ego *et al*. ont comparé les taux de PAG (au 10^e^ percentile) et de macrosomie (90^e^ percentile) entre les modèles M0, M1 (qui prend en compte le sexe), M2, (qui prend en compte le sexe et d´autres paramètres maternels) et d´autres courbes standardisées. Pour les poids maternels élevés, les courbes personnalisées donnent un taux élevé de PAG (12,9%) et faible de macrosomie (4,8%). À l´inverse, les courbes de poids de naissance de Leroy-Lefort *et al*. Audipog (non ajustée) et la courbe *in utero* du Collège Français d´Echographie Fœtale (CFEF) sous-estiment, selon les auteurs, la proportion des PAG (6,9%, 8,5%, 6,8% respectivement), par rapport aux modèles M1 ou M2 (10 à 11,9%) [18].

Francis *et al*. donnant l´exemple de l´Inde, notent un taux élevé de PAG selon *Intergrowth 21^st^* pour l´Inde (16,8%), représentant vraisemblablement, selon les auteurs, une variation physiologique due à l´origine ethnique et à la petite taille de la mère. D´après la courbe ajustée Gestation-related optimal weight (GROW), le taux de PAG est de 11,3% dans la même population [15]. Selon Ego *et al*. comparé au groupe des femmes donnant naissance à des enfants eutrophes selon les modèles M1 et M2, le groupe des nouveaux PAG du modèle M2 sont de mères multipares (79,4%), plus âgées (30,8 ± 5,2 ans), plus grandes (169,1 ± 6 cm) et corpulentes (76,3 ± 15,8 kg). Ces femmes sont 2 à 3 fois plus souvent hypertendues et grandes fumeuses (≥ 10 cigarettes/jour) (p < 0,0001). Ils ont conclu que ce groupe est celui dans lequel la proportion de patientes à haut risque de RCIU ou d´autres complications est la plus élevée (21,6% et 36,3%). À l´inverse, les mères de nouveau-nés PAG uniquement d´après le modèle M1 sont plus jeunes (28,3 ± 5,4 ans), souvent nullipares (79,2%), et de corpulence moindre (158,2 ± 5,2 cm et 50,8 ± 6,0 kg). Dans ce groupe, 70,1% des mères sont des femmes à bas risque [18]. Les enfants identifiés comme de nouveaux PAG par M2 ont, selon la même étude, une moins bonne adaptation à la vie extra-utérine (1,7% d´Apgar < 7 à 5 min versus 0,4%, p = 0,03) et sont plus souvent transférés en néonatologie (10,8 % versus 6,8 %, p < 0,01) [18].

Même si nos tests statistiques ne mettent souvent pas en évidence de différences statistiquement significatives au sein des sous-échantillons concernés, écueil certainement lié à la taille de ces derniers, la morbidité maternelle ainsi que la morbidité et mortalité périnatale est plus élevée dans le groupe d´enfants défini PAG uniquement par le modèle de Gardosi *et al*. Dans leur large cohorte, Francis *et al*. concluent également à une mortalité périnatale supérieur (2,4 contre 1,2%, RR = 1,9) et à une plus grande proportion de femmes à risques multiples à chez les nouveau-nés PAG uniquement par le modèle GROW [15].

## Conclusion

La courbe de croissance fœtale customisée de Gardosi *et al*. ajustée aux paramètres maternelles, classe plus de nouveau-nés PAG que la courbe standardisée de EPOPé M0 dans notre population d´Afrique Subsaharienne. La courbe ajustée classe les nouveau-nés PAG indifféremment de l´IMC. Enfin en cas de pathologie maternelles et néonatale, la courbe personnalisée identifie plus de nouveau-nés PAG. Il est important de privilégier la sensibilité d´un test dans cette situation et donc les courbes customisées peuvent être utilisées, en plus des courbes standardisées, afin d´identifier les nouveau-nés à risque et de planifier une prise en charge.

### Etat des connaissances sur le sujet

- Les courbes de croissance permettent de diagnostiquer les nouveau-nés PAG qui présentent une morbidité et une mortalité plus élevées.

### Contribution de notre étude à la connaissance

- Les courbes customisées notamment celle de Gardosi et al. sont plus adaptées à notre population afin d´identifier les nouveau-nés PAG et de planifier ainsi leur prise en charge.

## Remerciements

A tous les internes et médecins inscrits au Diplôme d´Etudes Spécialisées de Gynécologie-Obstétrique qui ont participé au maintien et à l´exhaustivité de la base de données *e_périnatal*.

## Annexe

**Annexe 1**: équations prédisant les poids aux 3^e^, 10^e^, 50^e^, 90^e^et 97^e^percentiles
